# Identifying normal mammograms in a large screening population using artificial intelligence

**DOI:** 10.1007/s00330-020-07165-1

**Published:** 2020-09-02

**Authors:** Kristina Lång, Magnus Dustler, Victor Dahlblom, Anna Åkesson, Ingvar Andersson, Sophia Zackrisson

**Affiliations:** 1 Diagnostic Radiology, Department of Translational Medicine, Lund University, Inga Maria Nilssons gata 47, SE-20502 Malmö, Sweden; 2 Unilabs Mammography Unit, Skåne University Hospital, Jan Waldenströms gata 22, SE-20502 Malmö, Sweden; 3 Radiology Department, Skåne University Hospital, Inga Maria Nilssons gata 47, SE-20502 Malmö, Sweden; 4 Clinical Studies Sweden – Forum South, Skåne University Hospital, Lund, Sweden

**Keywords:** Mammography, Mass screening, Breast cancer, Artificial intelligence

## Abstract

**Objectives:**

To evaluate the potential of artificial intelligence (AI) to identify normal mammograms in a screening population.

**Methods:**

In this retrospective study, 9581 double-read mammography screening exams including 68 screen-detected cancers and 187 false positives, a subcohort of the prospective population-based Malmö Breast Tomosynthesis Screening Trial, were analysed with a deep learning–based AI system. The AI system categorises mammograms with a cancer risk score increasing from 1 to 10. The effect on cancer detection and false positives of excluding mammograms below different AI risk thresholds from reading by radiologists was investigated. A panel of three breast radiologists assessed the radiographic appearance, type, and visibility of screen-detected cancers assigned low-risk scores (≤ 5). The reduction of normal exams, cancers, and false positives for the different thresholds was presented with 95% confidence intervals (CI).

**Results:**

If mammograms scored 1 and 2 were excluded from screen-reading, 1829 (19.1%; 95% CI 18.3–19.9) exams could be removed, including 10 (5.3%; 95% CI 2.1–8.6) false positives but no cancers. In total, 5082 (53.0%; 95% CI 52.0–54.0) exams, including 7 (10.3%; 95% CI 3.1–17.5) cancers and 52 (27.8%; 95% CI 21.4–34.2) false positives, had low-risk scores. All, except one, of the seven screen-detected cancers with low-risk scores were judged to be clearly visible.

**Conclusions:**

The evaluated AI system can correctly identify a proportion of a screening population as cancer-free and also reduce false positives. Thus, AI has the potential to improve mammography screening efficiency.

**Key Points:**

*• Retrospective study showed that AI can identify a proportion of mammograms as normal in a screening population.*

*• Excluding normal exams from screening using AI can reduce false positives.*

## Introduction

Breast cancer screening with mammography is one of the largest secondary prevention programmes in medicine and is widely implemented in high-income countries [1, 2]. The European screening guidelines recommend double-reading in order to increase screening sensitivity [3]. The double-reading procedure may be difficult to accomplish due to a shortage of radiologists specialising in breast imaging in many countries [4]. Daily reading of a large number of normal mammograms is a tedious task reducing the attractiveness of the field. Double-reading can also increase the risk of false positives [5]. Experiencing a false-positive screening can result in breast cancer–specific anxiety that can last up to 3 years [6]. Also, women with a false-positive screening are less likely to return for subsequent screening rounds [6].

The advent of artificial intelligence (AI) in medical imaging could, however, provide means to improve the efficiency of mammography screening by reducing the need of human readers and avoid false positives [7]. Recent studies have shown that AI can reach a similar or even higher accuracy in reading mammograms than human readers [8–11], as well as in improving reader performance when used as a decision support [10, 12]. Many of these studies were performed on enriched datasets and studies on AI performance on pure screening data are still scarce.

The aim of this study was to evaluate the potential of a commercially available AI system to identify normal mammograms in a breast cancer screening population, thereby reducing workload related to the radiologists’ screen-reading and false positives. In addition, the characteristics of screen-detected cancers that were missed by the AI system were assessed.

## Material and methods

The study was approved by the Regional Ethics Review Board (2009/770) and the Swedish Ethical Review Authority (2018/322). Informed consent was obtained.

### Study population

A consecutive subcohort from the prospective population-based Malmö Breast Tomosynthesis Screening Trial [13], consisting of 9581 women aged 40–74 (mean age 57.6 ± 9.5) with double-read screening mammograms, was included. The screening intervals were 1.5 years until the age of 55 and thereafter biennial screening. The subcohort consisted of a consecutive inclusion of trial participants with two-view digital mammograms (Mammomat Inspiration, Siemens Healthcare GmbH) for which both raw and processed imaging data were available (February 2012 until May 2015). Of the 9581 women, 255 were recalled (recall rate 2.7%) resulting in 68 screen-detected cancers (cancer detection rate 7.1/1000) and 187 false positives. Ground truth was based on histology of surgical specimen or core-needle biopsies and with a cross-reference to a regional cancer register. A normal mammogram was defined as free of screen-detected cancer. Participants in the Malmö Breast Tomosynthesis Screening Trial were also examined with tomosynthesis, but for the purpose of this study, only the independent mammography reading results were taken into account.

### AI-derived risk scores

All mammograms were analysed with a commercially available automatic breast cancer detection AI system based on deep convolutional neural networks (Transpara v.1.4.0, ScreenPoint Medical). The AI system assigns screening exams a risk score of 1–10, with 10 indicating the highest probability of malignancy [14–19]. The cancer risk scores are derived from a two-step process in which a traditional set of image classifiers and deep convolutional neural networks are first used to identify suspicious lesions, i.e. calcifications and soft tissue masses, which are further classified using a combination of another set of deep convolutional neural networks. The local detections are then combined into a risk score for the whole exam. The risk scores are calibrated to yield approximately one-tenth of screening mammograms in each category. In this study, we defined low-risk scores as 1–5 and high-risk scores as 6–10.

This version of the AI system was trained and validated using a database of about 180,000 normal and 9000 abnormal mammograms from four different vendors [11]. The mammograms used in this study had not been used in prior training or validation of the AI system.

### Review of AI-missed cancers

A consensus panel of three breast radiologists (each with > 7 years of experience) assessed the radiographic appearance, size, and visibility of cancers, as well as the mammographic density of mammograms with screen-detected cancers that were assigned low-risk scores. These cancers could be considered missed by the AI system. The consensus panel had access to all clinical information including pathology reports. The radiographic tumour appearance and mammographic density for the whole study population were previously assessed, as described in a prior publication [13].

### Statistical analysis

The effect of AI in screening was analysed by quantifying the number and frequencies of screen exams, screen-detected cancers, and false positives for the different risk scores. Wald confidence intervals (CI) were defined for the reduction of screen exams, screen-detected cancers, and false positives with low-risk scores, and calculated at the 95% confidence level. Calculations were performed in R (version 3.5.1, www.r-project.org). Furthermore, the distribution of risk scores in relation to tumour biology was assessed. Number and frequencies were used to present population characteristics, tumour biology, and radiographic appearance.

## Results

**Fig. 1:**
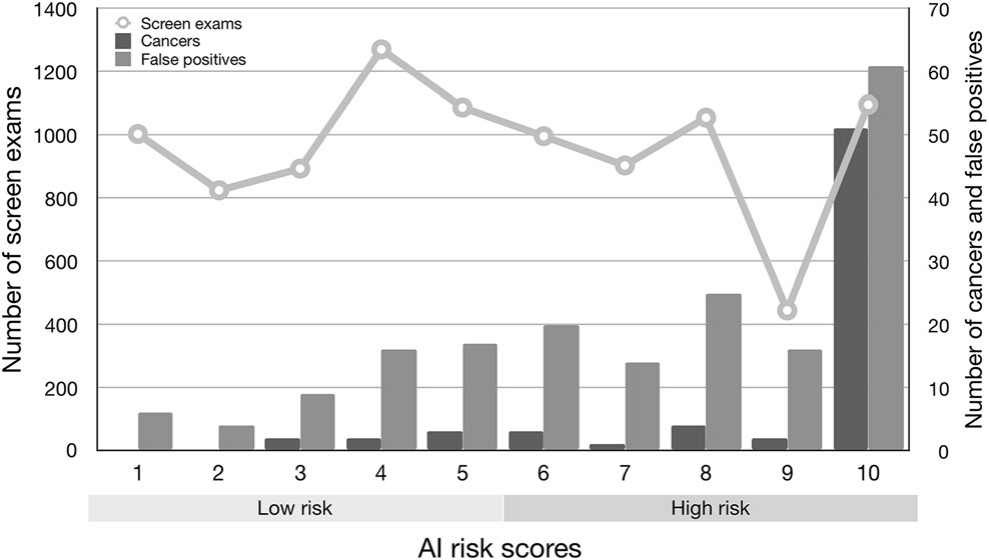
Distribution of AI risk scores for all mammography-screen exams, screen-detected cancers, and false positives

**Fig. 2:**
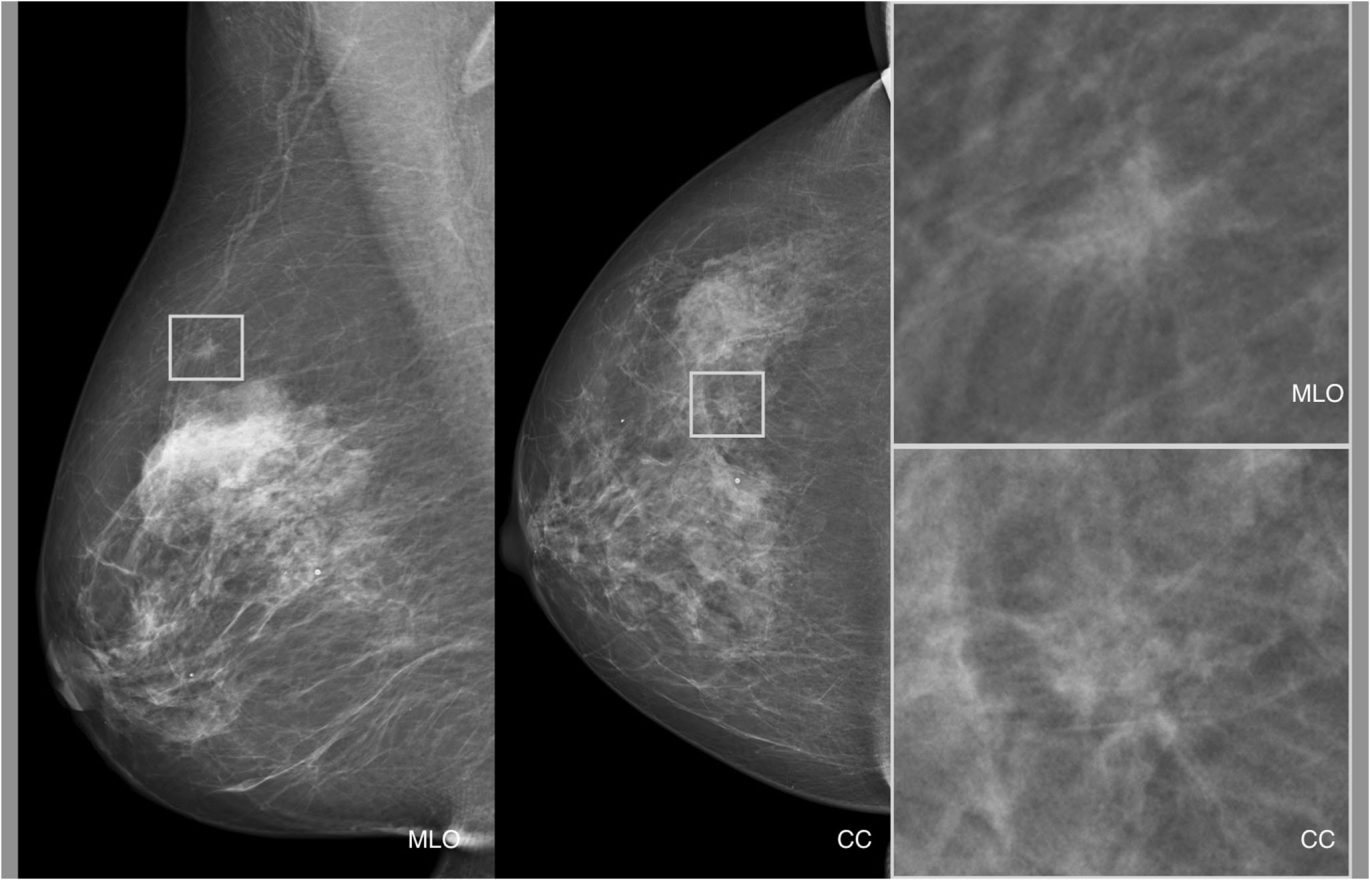
A cancer missed by the AI system. A 7-mm-large invasive tubular cancer (grade 1) with the radiographic appearance of a spiculated mass that was categorised with an AI risk score of 3. MLO, mediolateral oblique view; CC, craniocaudal view

The distribution of risk scores for all mammograms, screen-detected cancers, and false positives is shown in Fig. 1. The cancer incidence in mammograms with low- and high-risk scores was 1.4/1000 and 13.6/1000, respectively. If mammograms with a risk score of 1 and 2 were to be excluded, 1829 (19.1%; 95% CI 18.3–19.9) normal exams could be removed, including 10 (5.3%; 95% CI 2.1–8.6) false positives, without missing a single cancer (Table 1). Half (53.0%, 95% CI 52.0–54.0) of the screen exams had low-risk scores (≤ 5). If these were to be excluded from screen reading performed by radiologists, seven (10.3%; 95% CI 3.1–17.5) cancers would have been missed, and 52 (27.8%; 95% CI 21.4–34.2) false positives would have been avoided. All seven cancers with low-risk scores were invasive (Table 2), of which three were small (≤ 7 mm), low-grade invasive tubular carcinomas, i.e. tumours with excellent prognosis [19]. On the other hand, three cancers, two ductal and one lobular type, were large (20 mm), one of which was histologic grade 3, i.e. of less-favourable prognosis. The radiologists’ consensus panel judged all cancers, except one, to be clearly visible (Fig. 2). The latter was a 20-mm-large mammographically occult invasive ductal carcinoma that was recalled due to an imaging finding of a pathologically enlarged lymph node. Six of the cancers had a radiographic appearance of a spiculated mass. All, except one, of the women with AI-missed cancers had dense breasts (Breast Imaging Reporting and Data System-category C and D [21]).

**Table 1:** The accumulated effect of excluding mammography screen exams assigned low AI risk scores

Risk scores	Screen exams, *n* (% [95% CI])	Cancers, *n* (% [95% CI])	False positives, *n* (% [95% CI])
1	1004 (10.4 [9.9–11.1])	0	6 (3.2 [1.5–6.8])
1–2	1829 (19.1 [18.3–19.9])	0	10 (5.3 [2.9–9.6])
1–3	2723 (28.4 [27.5–29.3])	2 (2.9 [0.8–10.1])	19 (10.2 [6.6–15.3])
1–4	3994 (41.7 [40.7–42.7])	4 (6.7 [2.3–14.2])	35 (18.7 [13.8–24.9])
1–5	5082 (53.0 [52.0–54.0])	7 (10.3 [5.1–19.8])	52 (27.8 [21.9–34.6])
Total (1–10)	9581 (100.0 )	68 (100.0)	187 (100.0)

**Table 2:** Histological characteristics of screen-detected cancers categorised with low- and high-AI-risk scores (low scores = 1–5 and high scores = 6–10)

	Low risk, *n* (% [95% CI])	High risk, *n* (% [95% CI])
Histologic type		
IDC	3 (42.9 [6.2–79.5])	30 (49.2 [37.1–61.4])
ILC	1 (14.3 [2.6–51.3])	10 (16.4 [9.2–27.6])
Tub	3 (42.9 [6.2–79.5])	7 (11.5 [5.7–21.8])
DCIS	0	11 (18.0 [10.4–29.5])
Other*	0	3 (4.9 [1.7–13.5])
Total	7 (100.0)	61 (100.0)
Histologic grade invasive cancers		
Grade 1	4 (57.1 [25.0–84.2])	20 (40.8 [28.2–54.8])
Grade 2	2 (28.6 [8.2–64.1])	23 (46.9 [33.7–60.6])
Grade 3	1 (14.3 [2.6–51.3])	6 (12.2 [5.7–24.2])
Total	7 (100.0)	49 (100.0)

*IDC* invasive ductal cancer, *ILC* invasive lobular cancer, *Tub* invasive tubular cancer, *DCIS* ductal carcinoma in situ

*e.g. papillary carcinoma, apocrine tumour

### AI performance in relation to tumour biology

**Fig. 3:**
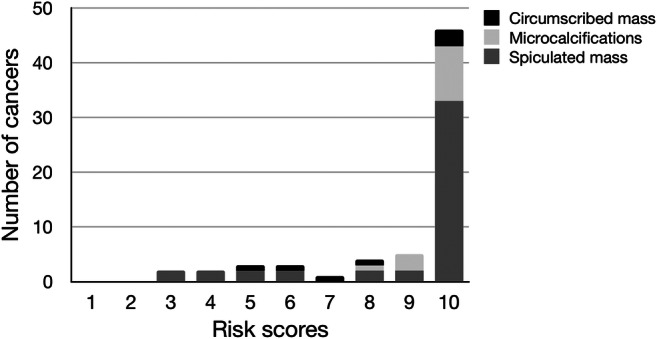
Distribution of AI risk scores in relation to radiographic appearance of screen-detected cancers. Three cancers are not included in the analysis (women recalled due to enlarged lymph node or due to symptoms)

As shown in Table 2, the most common type of screen-detected cancers was an invasive ductal carcinoma. The majority of mammograms with invasive ductal carcinomas were classified with high-risk scores. Notably, 10 out of 11 mammograms with invasive lobular cancers, a cancer type that is known to sometimes have a subtle radiographic appearance, were also classified with high-risk scores. Furthermore, high-risk scores were assigned to all cancers with calcifications as the dominating radiographic feature (Fig. 3). The majority (10/14) of these were ductal carcinoma in situ. Finally, all but one of the seven high-grade cancers had a risk score of 10.

## Discussion

The present study aimed to assess whether AI could identify normal exams in mammography screening. We found that with AI, every fifth mammogram could be excluded from screen reading performed by radiologists without missing cancers, and at the same time a number of false positives could be avoided. Consequently, radiologists’ workload and costs related to screen reading and false positives could potentially be reduced. Considering that the double-reading procedure is practiced in many screening programmes, especially in Europe [22], the saving could be substantial. In this specific Swedish screening setting with low recall rates (2.6%), the reduction of false positives was small. It is fair to assume that the reduction of false positives could be greater in a setting where the recall rates are higher, such as in the USA [23, 24]. The majority of the false-positive mammograms had high-risk scores, reflecting the fact that both human readers and AI found suspicious features in the same image.

The size of the reduction of screen exams from radiologists’ reading also depends on whether the trade-off in terms of a slight reduction of sensitivity could be considered acceptable. If we would exclude mammograms with low-risk scores (half of all screen exams), 28% of the false positives could be avoided. This does not seem acceptable since 10% of the cancers would have been missed. Since half of the AI-missed cancers were indolent cancers, i.e. low-grade invasive tubular cancers, the trade-off might still be considered. We have to keep in mind that the results are point estimates with mostly broad confidence intervals; the percentage of missed cancers may be as few as 3% and as many as 18%. The magnitude of normal exams identified in this study was similar to the results presented by Rodriguez-Ruiz et al using the same AI system, but on a study population with both clinical and screening mammography exams [25], and by Yala et al using a different AI system than the one used in this study, on a large screening data set [26].

We were not able to unravel why the AI system missed cancers, since all but one had a clearly visible lesion in the breast. However, since the cancers were visible, there seems to be room for improvement of the AI system. We can expect that AI algorithms improve over time with further training; in fact, the AI system used in this study has evolved from version 1.4.0 to 1.6.0. With this improvement, we could potentially, by excluding mammograms with low-risk scores, safely automate a substantial part of the screen reading. The effect on interval cancers, i.e. false negatives, has not been included in the present study due to small numbers, but is currently being investigated in a larger cohort. However, in the cohort used in this study, no interval cancer was later diagnosed among women in AI risk group 1 or 2. Still, the medico-legal and ethical challenges using AI as a stand-alone reader in screening when a cancer is missed are expected to be considerable [7]. To automatically discard low-risk exams from human reading might therefore not be possible. The risk scores could, however, potentially be used to address the screen-reading workload by triaging exams to either single or double reading.

In this study, the AI system was shown to be especially sensitive in detecting microcalcifications, which is a common, and often the single, radiographic feature of ductal carcinoma in situ*.* The ductal carcinoma in situ lesions all received high AI risk scores (i.e. score 6–10 of which 55% received a score of 10). This implies that using this AI system in screening is likely to maintain or increase the detection rate of in situ cancers, hence possibly adding to overdiagnosis [27]. On the other hand, of the cancers that were missed by AI, three out of seven were small, low-grade invasive tubular cancers, which in the light of overdiagnosis might not necessarily be a drawback [28]. Studies with other AI vendors have shown varying results; the sensitivity for calcifications can increase with the assistance of AI [10] or that AI seems to be more sensitive to invasive than in situ cancers [8].

The generalizability of these results is subject to certain limitations. The study data was derived from a single-screening centre with specific conditions, e.g. an urban Swedish population, experienced breast radiologists, the use of the double-reading procedure, and using only one mammography and AI vendor combination. Therefore, the results need to be validated retrospectively on other screening data sets, and subsequently in a prospective trial. Another aspect is how well radiologists will perform using the AI system as decision support rather than as an independent pre-sorting method as is proposed in this study. It is reasonable to assume that the radiologists would be influenced by the knowledge of the risk scores in a prospective setting, affecting both sensitivity and specificity [29]. Another limitation of this study was the small sample size of cancers that did not allow for any subgroup analyses, besides descriptive statistics. Furthermore, the study population was based on a prospective screening trial comparing tomosynthesis with mammography [13], but the scope of this study was limited to evaluating the mammography results. In the trial, additional cancers were detected with breast tomosynthesis and the performance of the AI system on the corresponding mammogram is currently being investigated, as well as the performance in mammography in relation to breast density.

In conclusion, this study has shown that AI can correctly identify a proportion of a screening population as cancer-free and also reduce false positives. Thus, AI has the potential to improve the mammography screening efficiency by reducing radiologists’ workload and the negative effects of false positives.

## Abbreviations

AI: Artificial intelligence
CI: Confidence interval
